# Electrochemical impedance spectroscopy as a micropropagation monitoring tool for plants: A case study of tamarillo *Solanum betaceum* callus

**DOI:** 10.1016/j.isci.2025.111807

**Published:** 2025-01-14

**Authors:** André Caeiro, Jorge Canhoto, Paulo R.F. Rocha

**Affiliations:** 1 Centre for Functional Ecology, Laboratory Associate TERRA, Department of Life Sciences, University of Coimbra, 3000-456 Coimbra, Portugal

**Keywords:** Biotechnology, Plant biology, Plants, Engineering

## Abstract

Tamarillo is an economically important species that has been extensively studied in terms of *in vitro* morphogenesis and micropropagation techniques, including somatic embryogenesis. *Callus* cultures are used to characterize plant growth and differentiation as well as the production of secondary metabolites. In all cases, real-time screening methods to characterize cellular growth remain poorly explored. Here, we show that electrochemical impedance spectroscopy (EIS) enables non-invasive and real-time monitoring of *callus* growth and cytological changes. A non-embryogenic *callus* line derived from leaf explants of tamarillo was used. The *callus* was tested in both standard culture medium containing the auxin picloram and in auxin-free medium. The low frequency impedance modulus and the equivalent circuit charge transfer resistance are found to effectively translate real-time cellular growth and microstructural deformations which have been benchmarked with light and scanning electron microscopy and mass measurements. EIS therefore emerges as a micropropagation monitoring technique in plant biotechnology.

## Highlights

• EIS enables non-invasive monitoring of *callus* growth and cytological changes
• EIS translates in real-time cellular microstructural deformations in *Callus* cultures
• EIS emerges as a micropropagation monitoring technique in plant biotechnology

## Graphical abstract

**Figure:**
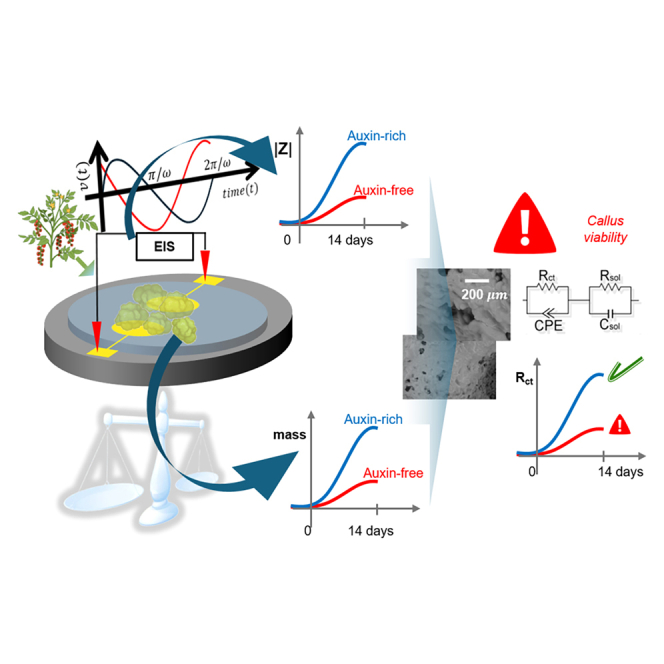

## Introduction

Global crop production strongly relies on high-quality propagating plant materials and methodologies to mitigate irregular production or debilitating crop diseases triggered by unpredictable pathogens or climate changes.^1^^,^^2^ For instance, protection of vegetatively propagated plants against viruses and viroids relies on invasive laboratory-dependent tissue culture methods including somatic embryogenesis (SE). This micropropagation method warrants sanitation candidate materials but through a high price tag associated with highly specialized technical personnel and laboratory infrastructures.^3^^,^^4^ Thus, there is a major need in plant biotechnology for rapid, affordable, reproducible, and non-destructive methodologies for assessing cellular differentiation, growth, and multiplication.

Somatic embryogenesis is a process in which somatic cells, under specific stimuli, develop into somatic embryos identical to their zygotic counterparts. Somatic embryo undergoes the normal developmental stages and can eventually germinate into a plant.^5^ SE has been extensively reported and optimized for several explants and culture conditions,^6^ particularly in *Solanum betaceum* Cav. (tamarillo),^7^ an emerging subtropical non-climacteric fruit, capable of uninterrupted production and offering highly nutritious fruits and derived products.^8^^,^^9^^,^^10^ In this species, using explanted leaf segments cultured in an auxin-rich (picloram) medium, supplemented with high (26 mM) concentrations of sucrose, non-embryogenic callus can be produced. Non-embryogenic *callus* (NEC), a non-embryogenic phenotype^11^^,^^12^ have multiple applications in biotechnology, such as the production of hydrolytic enzymes or secondary metabolites.^13^ These c*allus* can be sub-cultured for several years in the same auxin-rich medium.^14^ Auxins are an extremely important class of plant growth regulators (PGRs) that influence a wide variety of physiological processes, such as tropisms, embryogenesis, cellular division, and several relevant biotechnological processes.^15^^,^^16^ In the field of SE, for example, both endogenous and externally applied synthetic auxins are fundamental in most induction protocols.^17^

The NEC *callus* cultured in auxin rich medium has been described as an efficient source for plant cell suspension (PCS) cultures.^13^ Because of the specific cellular characteristics and plant biochemistry and molecular biology, PCS based methodologies show potential in several applications, namely pharmaceutical production, acquisition of secondary metabolites, and expression of heterologous proteins.^18^^,^^19^^,^^20^

The successful biotechnological application of plant cell suspensions is advantageous in several fields, namely recombinant protein production^21^ and is highly dependent on effective screening techniques. Several techniques, such as wet/dry weight measurement, packed cell volume occasionally couple with morphology, or viability assays have been described and employed.^22^^,^^23^ However, these techniques are in general destructive, laborious, commonly not suitable for real-time application, and occasionally prone to inconsistent measurements, particularly in terms of viability.^24^

Electrochemical impedance spectroscopy (EIS) is a non-invasive tool valuable to extract real-time comprehensive information related to multiple physical/chemical processes by determining their characteristic frequency response. EIS is particular useful to extract and determine the composition and conductivity of electrolyte solutions, the charging and discharging of the electric double layer in electrode/electrolyte interfaces and the kinetics of an electrode charge-transfer reaction, among others.^25^ Typically, a small voltage signal across the electrodes in an electrochemical cell is applied and the generated current signal is measured.^26^ The frequency of the excitation voltage signal is varied to produce an impedance spectrum.^27^^,^^28^ Due to the simplicity of an EIS experimental setup and accurate readouts, EIS has been used as a diagnosis technique for a myriad of applications, from lithium-ion batteries,^29^ organic devices,^30^ and electrochemical sensors^26^ to probing and modeling neuronal adhesion and differentiation.^31^ Surprisingly, despite the non-invasive, real-time, and portability offered by of the shelf EIS systems, the use of EIS in plant cell culture or in the characterization of cell growth and differentiation has been scarce.^32^ Here, we employ EIS to probe, in real-time, the development of a non-embryogenic *callus* line derived from leaf explants on circular Au electrodes under both auxin-rich and auxin-free semisolid media. We selected the non-embryogenic *S. betaceum* as a model for biotechnological applications directly involved in cellular development. The kinetics of impedance change underlying the cellular development are benchmarked with standard mass measurements, mathematical and equivalent circuit modeling, and microscopy.

## Results

### Somatic embryogenic induction and characterization of callus morphology

Before the beginning of experiments, the non-embryogenic phenotype was confirmed by seeding NEC tissue on auxin-free maturation medium with a reduced sucrose content. Under these conditions few to none somatic embryos were visualized, which confirmed the lack of embryogenic competence.

**Figure 1:**
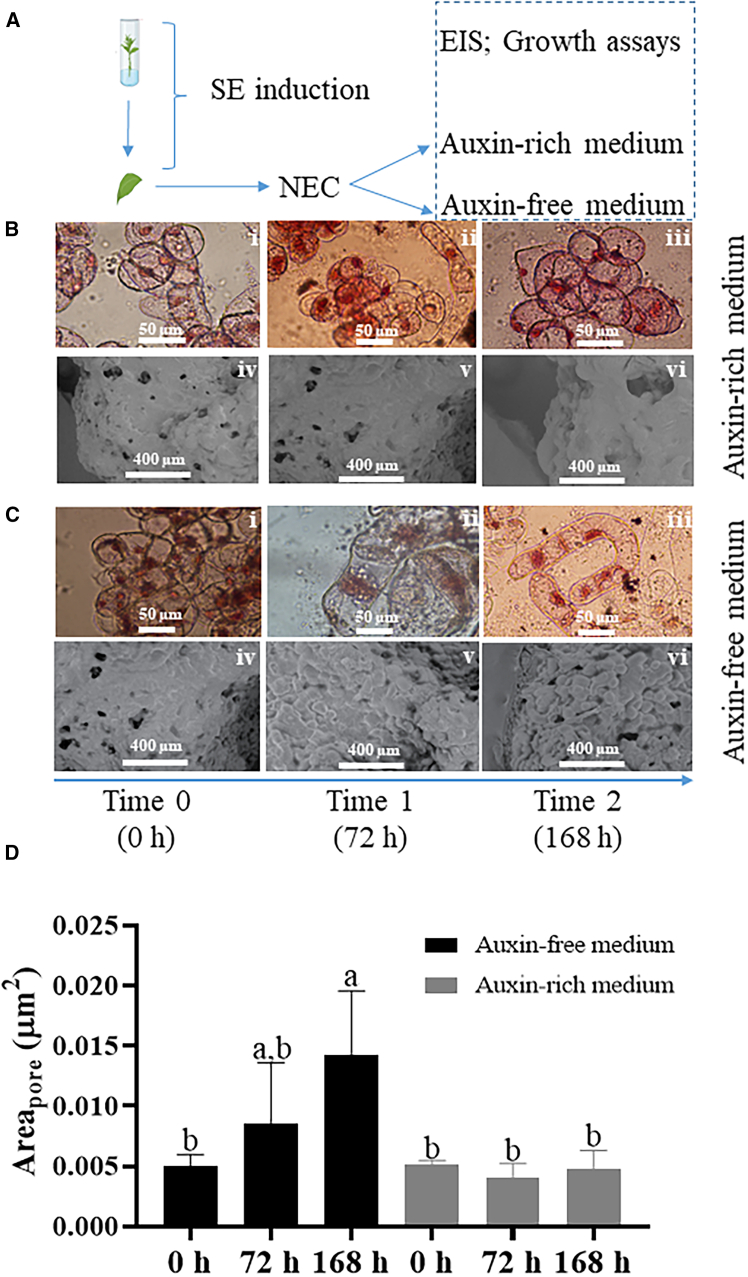
*Callus* morphology (A) General scheme of somatic embryogenic induction and *callus* growth. (B) NEC *callus* in auxin-rich medium. (C) NEC *callus* in auxin-free medium. In each group, bright-field microscopy images are on the first raw (scale bar represents 50 μm) and SEM images on the second (scale bar represents 400 μm). (D) Average porous diameter over time (hours) on auxin-free (black bars) and auxin-rich (gray bars) medium. Different letters represent significant different values by Tukey’s test (*p* < 0.05).

The induction protocol is shown in Figure 1A. To expand *callus* mass, subcultures were made. The subcultures were then grown on auxin-rich and auxin-free medium as represented in the diagram of Figure 1A. In auxin-rich medium, the cell morphology over time remained relatively unchanged, with mostly circular cells appearing in small clusters. This was observed by the bright-field images shown in Figure 1B, i–iii. A few distinct cellular morphologies characterized by an elongated cytoplasm (Figure 1B, ii) were also sporadically observed. In terms of SEM observation, (Figure 1B, iv–vi) the overall morphology of the clusters also remained largely unchanged with a smooth surface. A few pore-like structures were observed in the NEC surface. On the other hand, in auxin-free medium, cellular morphological changes were observed (Figure 1C). In the optic observations, distinct morphologies were much more common, in comparison to NEC growing in auxin-rich medium. Cells often display an elongated cytoplasm (Figure 1C, ii) or a distinctly wider nucleus (Figure 1C, iii). In the SEM micrographs (Figure 1C, iv–vi), the surface of the clusters appeared much less uniform over time. Yet, the area of the pore-like structures systematically increased over time (Figure 1D) reaching about 0.02 μm^2^ at 168 h. Changes in the area of the pore-like structures, in auxin rich medium, were not statistically significant.

**Figure 2:**
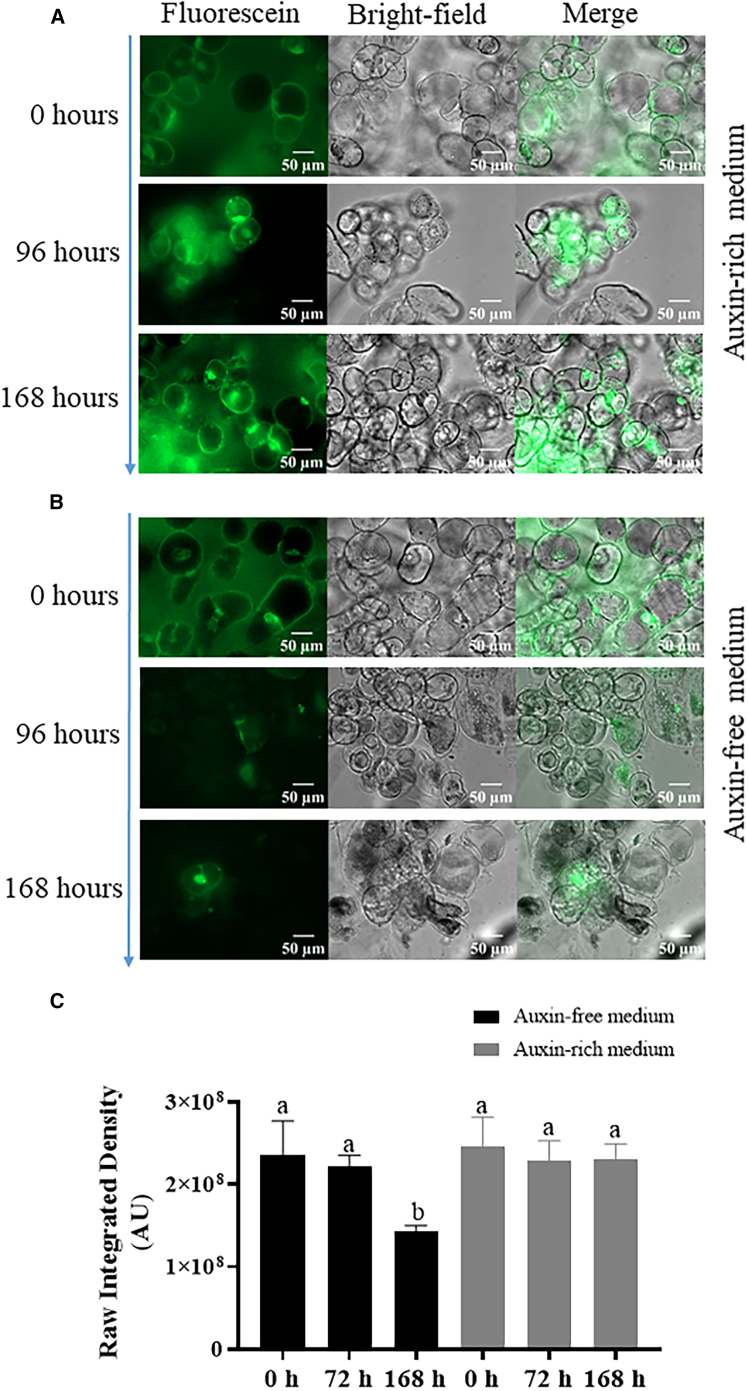
Cell viability (A and B) Intracellular green-labeling indicates viability (A) NEC *callus* in auxin-rich medium and (B) NEC *callus* in auxin-free medium. In each group fluorescence is on the first column, bright-field on the second and merged fields on the third. Bar represents 50 μm. (C) Raw integrated density on auxin-free (black bars) and auxin-rich medium (gray bars). Different letters represent significant different values by Tukey’s test (*p* < 0.05).

To ascertain viability as a function of time, we performed fluorescence measurements of *callus* grown in both media and analyzed the raw integrated density (RawIntDen). In auxin-rich medium (Figure 2A), the *callus* maintained a good viability over time, namely at 0, 72, and 168 h. On the other hand, when *callus* was grown on auxin-free medium (Figure 2B), a decrease was observed in the median RawIntDen from 2.5 × 10^8^, 2.2 × 10^8^, and 1.4 × 10^8^ at 0, 72, and 168 h, respectively (Figure 2C). Interestingly, we note the relative difference in the average pore area (Figure 1D), from 72 to 168 h increases 40%. This agrees with the relative difference in RawIntDen, in the same time interval, which diminishes 36%.

### Cell adhesion to Au electrodes decreases low-frequency capacitance and increases impedance

**Figure 3:**
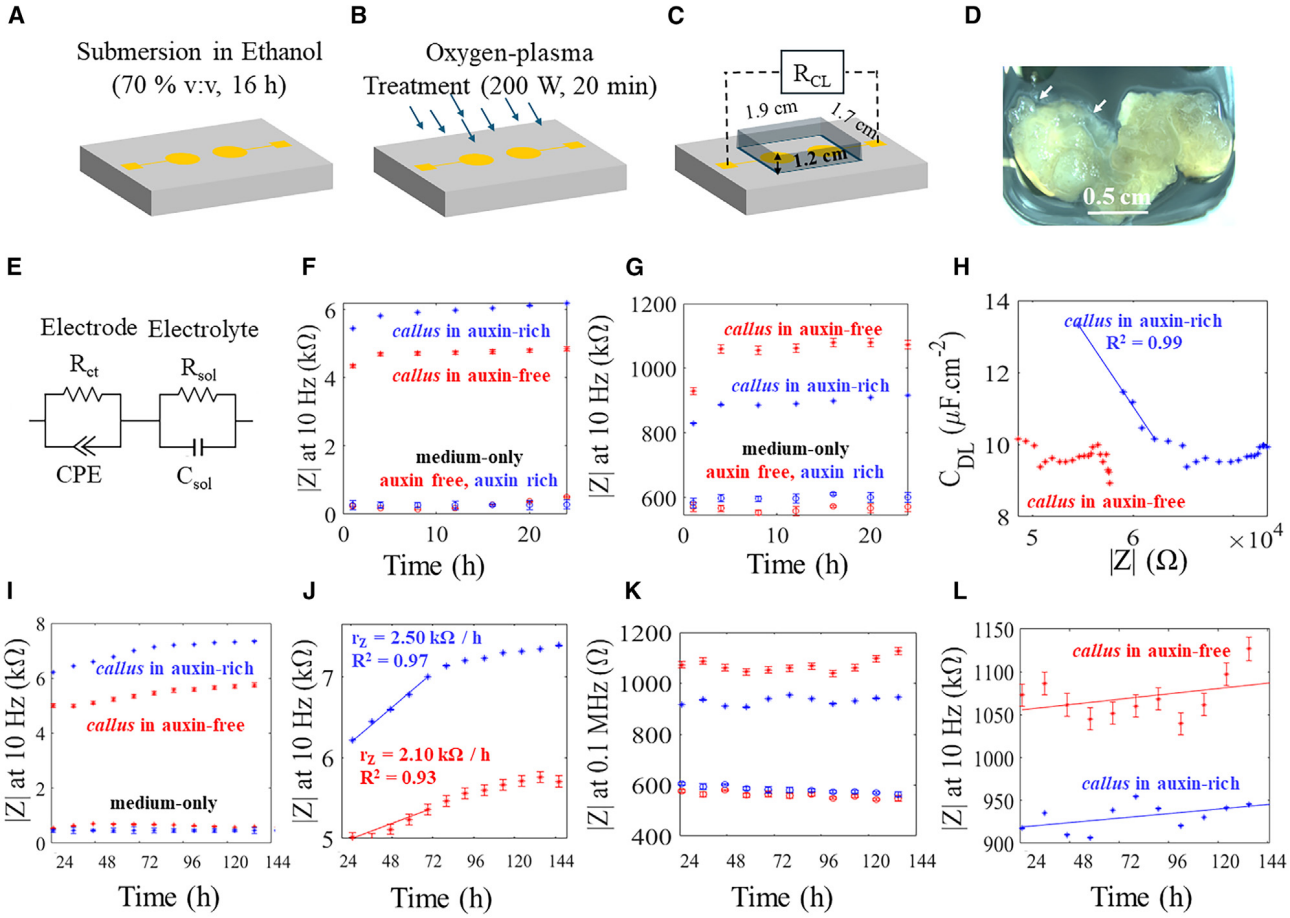
Impedance setup and measurements over time (A and B) (A) The transducer was cleaned and (B) exposed to oxygen O_2_-plasma immediately before *callus* seeding and EIS recordings. (C) Illustration of connections to the measuring instrumentation. (D) *Callus* on auxin-rich medium on the circular electrode. (E) Equivalent double RC circuit. (F) Impedance at low frequency (10 Hz) during the first 24 h. (G) Impedance at high frequency (0.1 MHz) during the first 24 h. (H) Impedance modulus as a function of $C_{dl}$ for both induction media, with *callus* in auxin-rich media exhibiting a high correlation (R^2^ = 0.99) in the line drawn. (I) Impedance modulus at 10 Hz, after 24 h. (J) Zoom in of (I) with rate of increase in impedance extracted from the first 96 h. (K) Impedance modulus after 24 h at 0.1 MHz. (L) Zoom in of (K). Data are presented as mean ± SD (*n* = 3).

Cells and culture medium were measured at regular 1-h intervals for 7 days using EIS (Figures 3A–3C). *Callus* development on the electrode surface was observed, with *callus* expanding to the surrounding area and a mucilaginous layer visible on the edges (Figure 3D). The measured EIS data were analyzed, and equivalent circuit modeling made on Zview 2. The equivalent circuit shown in Figure 3E comprises the equivalent parallel capacitance in parallel with a charge transfer resistance $R_{ct}$ and in series with a spreading resistance ($R_{sol}$) which represents the culture medium resistivity. The capacitance, due to the electrode-electrolyte interface, is best modeled by as constant phase element (CPE) whose impedance is given by:

(Equation 1) $$\begin{array}{c} Z_{CPE}\left(\omega \right)=\frac{1}{Q{\left(j\omega \right)}^{n}} \end{array}$$

Where n is a rational exponent that varies between 0 and 1 and Q is a frequency independent parameter. The double layer capacitance, $C_{dl}$, can be estimated with the following Equation 2 ^31^^,^^38^:

(Equation 2) $$\begin{array}{c} C_{dl}=\frac{{\left({QR}_{ct}\right)}^{\frac{1}{n}}}{R_{ct}} \end{array}$$

The impedance modulus (from herein referred to as impedance) at low (10 Hz) and high (0.1 MHz) frequencies was analyzed over time. In the first 24 h, the impedance at 10 Hz, increases in the first 6 h and then stabilizes (Figure 3F). The impedance increase of over 3 kΩ is due to cellular adhesion. The first 24 h also show an increase in high frequency impedance although less pronounced, of over 200 *Ω* as shown in Figure 3G. The significant increase over time in the low frequency impedance are mostly due to the decrease in the equivalent parallel capacitance as seen by the linear relation between $C_{dl}$ and impedance, particularly in the first 72 h of *callus* growing in auxin-rich medium (Figure 3H). Increasing cell-layer thickness seems to decrease the measured capacitance and increase the measured low-frequency impedance. In Figure S1, the low frequency impedance, phase and capacitance as a function of frequency are shown for auxin-rich and auxin-free medium, at 0 h.

### Low frequency impedance correlates with cellular growth over time

We noted a clear difference in the measured impedance with cells containing medium comparable to their respective medium-only control. The low frequency impedance recordings, from 24 to 144 h, show an exponential increase using both culture mediums (Figure 3I). The rate of increase in impedance, *r*_*Z*_, (Figure 3J), describes a larger impedance change in *callus* cultures growing in auxin-rich medium of approximately 38 *Ω*/day over an average of 10 *Ω*/day of *callus* cultures growing in auxin-free medium. At 0.1 MHz, (Figure 3K) the time evolution of impedance in both media barely changed with a minor increase of 150 *Ω* over the full length of the experiment (Figure 3L).

**Figure 4:**
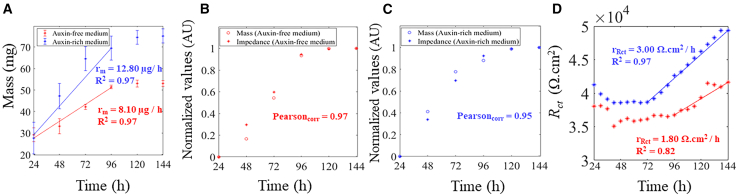
Growth parameters (A) Mass values over time (results are presented as mean ± SD (*n* = 3)). (B and C) Normalized mass and impedance values (10 Hz) over time. Pearson_corr_ is the Pearson correlation coefficient between normalized mass and impedance. (D) $R_{ct}$ parameter calculated from equivalent circuit models over time.

Conventional growth assays were conducted in parallel to the EIS measurements. In Figure 4A, in the first 96 h, a higher rate of increase in mass, *r*_*m*_ was determined when *callus* were grown in auxin-rich medium (12.8 *μ*g/h). On the other hand, when *callus* were grown in auxin-free medium a *r*_*m*_ of 8.1 *μ*g/h was found. Mass and impedance were then normalized and plotted as a function of time (Figures 4B and 4C). An excellent correlation between both impedance and mass was found with Person correlation values of 0.97 and 0.95, for auxin-free and auxin-rich medium, respectively). The charge transfer resistance, $R_{ct}$, extracted from *callus* in auxin-rich medium remained stable until 72 h, followed by an exponential increase from 72 to 144 h. A rate of increase in *r*_*Rct*_ of 3 *Ω* cm^*2*^*/*h is extracted. Parameters derived from equivalent circuit analysis, specifically $C_{dl}$, showed a decrease from 13.32 to 9.93 μF cm^−2^ (approximately 25%) in auxin-rich medium and from 10.15 to 8.73 μF cm^−2^ (approximately 14%) in auxin-free medium (Table S1). $R_{ct}$ extracted from *callus* in auxin-free medium remained stable for longer time (up to 96 h), and exhibited a slower exponential growth, of 40% less, in comparison to *callus* grown in auxin-rich medium. A lower rate of increase in *r*_*Rct*_ of 1.8 *Ω* cm^*2*^*/*h was determined.

## Discussion

### Cellular morphology is influenced by the culture medium

In tamarillo, NEC *callus* presents high proliferative rates and specific morphology but lacks embryogenic competence and will become necrotic if auxin supplementation is absent.^33^^,^^39^ In the present work, the NEC *callus* has shown a high rate of increase in mass and a relatively homogenous structure in the standard culture medium (supplemented with picloram and with a high concentration of sucrose). The rate of increase in mass and impedance were significantly reduced when the auxin was absent. In addition, we performed fluorescence experiments over time. The cellular hydrolysis of fluorescein diacetate into fluorescein is considered characteristic of viable cells and has been shown to provide a simple and rapid method for determination of cellular activity.^40^ Hence, by performing fluorescence experiments over time, in Figure 2B, we ascertained that the viability of *callus* growing in auxin-free medium is significantly reduced when compared to *callus* growing in auxin-rich medium (Figure 2A).

Although the presence of auxins in the culture medium is a widely used and considered of primordial importance for the SE process,^41^ some works have described other agents, such as high osmotic pressure by sucrose, non-metabolizable sugars or polymers can have a positive effect on embryogenic formation.^42^^,^^43^^,^^44^ Therefore, the high sucrose concentration in the culture medium may explain cellular behavior and effectively delay necrosis. Additionally, we noted the average pore size observed in *callus* growing in auxin-free medium increases over time (possibly due to necrosis), while remaining relatively constant on auxin-rich medium—an observation that is reflected in the exponential increase in low frequency impedance and $R_{ct}$, which we will now discuss in the following sections.

### Electrochemical impedance spectroscopy translates changes in cell morphology and microstructure

The Faradaic redox processes occurring at the electrode/electrolyte interface, during *callus* development or cell proliferation can be characterized by a frequency independent, charge transfer resistance, $R_{ct}$.^26^ In plants, $R_{ct}$ is sensitive to water distribution between intra and extracellular spaces and cell membrane integrity^45^^,^^46^ and has been used to assay root stress and nitrogen status in several important plant crops and weeds.^47^^,^^48^ In our case*,* the $R_{ct}$ measured when *callus* cultivated on auxin-free medium was different from that of *callus* cultivated in auxin-rich medium. The $R_{ct}$ first decreased over time until 48 h, for both auxin-rich and auxin-free media. Afterward, in auxin-rich medium, $R_{ct}$ was stable until 72 h with a *r*_*Rct*_ of 3 kΩ cm^2^/h. On the other hand, in auxin-free medium the stability period was larger, until about 96 h, and the exponential growth presented a much lower rate of increase, of 1.8 kΩ cm^2^/h. The increase in number of cells and concomitant production of extracellular matrix can contribute to Faradaic currents, which decreases the charge transfer resistance and polarizes the electrode. The $R_{ct}$ then changes its trajectory and increased exponentially beyond 72 h in auxin-rich medium and 96 h in auxin-free medium. This is possibly linked with the larger and more frequently observed pinholes (or pores) on cell tissue, facilitating access of electrolyte solution to the electrode. $R_{ct}$ can discriminate between physiological status in these recorded *callus*; however, the specific mechanisms underlying these changes would benefit from further investigation.

The capacitance increases residually with decreasing frequency because the double layer is not a linear capacitor but instead a constant phase element.^49^ At high frequency, the capacitance is dominated by the media, which is disregarded. A good fitting between measured and modeled capacitance was obtained for all measurements. The evolution of extracted $C_{dl}$, and all fitted parameters is shown in Table S1. An example of measured and modeled capacitance, at time 0, is shown in Figure S1.

Some studies have used electrolyte leakage as a assay to test membrane integrity,^50^ where the increase of ions in the culture medium either by loss of membrane integrity in the early necrotic states (in line with pores appearing at the membrane surface), or by changes to physiological state could be linked to auxin stimulation.^51^ Indeed, from the equivalent circuit analysis, we extracted the solution resistance, $R_{sol}$, as function of time and estimated a rate of change of 36.2 *Ω*/day for *callus* in auxin-free media, in comparison to 28 *Ω*/day in auxin-rich medium.

### EIS translates real-time structural developments of callus

Recordings with electrolyte media only showed stable values over the time period studied, which indicates that changes in impedance are correlated with cellular presence and development. In the period between 24 and 144 h, the impedance at low frequency, increased in both cultures. In auxin-rich medium, a higher *r*_*z*_ was determined as 2.5 k*Ω*/h. On the other hand, in auxin-free medium a lower *r*_*z*_ is determined, of 2.1 k*Ω*/h. An excellent correlation was found with impedance and mass, where a higher *r*_*m*_, of 12.8 μg/h, is found in cells cultured with auxin-rich medium (Figure 3A). The morphology and physiologic state of the *callus* appears to have influenced the impedance values. The low frequency impedance as a function of time accurately translated cellular growth where more compacted and metabolic active cells originated a higher impedance rate. In fact, the cellular wall seems to be quite important in this electrochemical translation. The cellular wall is a complex heterogeneous structure composed mainly of polysaccharides, and to a lesser extent, proteins, phenolic compounds, and water^52^^,^^53^ that maintains cellular integrity against the internal hydrostatic pressure, protects the cell from osmotic stress, provides several biochemical and transportation functions, and intervenes in other types of biotic and abiotic stress responses.^54^^,^^55^^,^^56^^,^^57^ On the other hand, the impedance at high frequencies showed an overall increase in the time interval studied with noticeable quasi-periodic oscillations. Circadian rhythms, namely in terms of gene expression, have been studied in plant culture^58^ and could be a possible explanation for the observed oscillations. In spite of this, the impedance at high frequency does not appear to be a good proxy parameter in our electrodes for cellular growth in tamarillo *callus*.

### Conclusions

The application of EIS to the monitorization and study of *callus* derived from an indirect somatic embryogenesis protocol has been demonstrated. The impedance modulus at low frequency correlates to cellular parameters, namely growth or cellular division. Cell adhesion and growth changes the cell-layer thickness above the Au surface which decreases the measured capacitance and increases the measured low-frequency impedance. The removal of the synthetic auxin picloram, used in the induction medium with the maintenance of a high sucrose supplementation, changed the *callus* phenotype in terms of rate of increase in mass and viability, which corresponded to a decrease in impedance rate larger than 15%. The relative change in impedance thus corresponds to the relative change in mass. The EIS analysis presented here informs, in real-time, on the cell-layer microstructure or porosity, leading to a decrease in charge transfer resistance rate larger than 40% when using the auxin-free induction medium. EIS could therefore contribute to advance biotechnology and clonal multiplication of plants by enabling future mechanistic comparisons between embryogenic and non-embryogenic *callus* and reveal details beyond mass or regular monitorization of *callus* development.

### Limitations of the study

Our study focused on non-embryogenic *callus* due to its several biotechnological applications. However, future studies should be based on mechanistic comparisons between embryogenic and non-embryogenic *callus* to further advance impedance-based monitorization strategies. Validation of this technique in *callus* derived from other plants should also be performed.

## Resource availability

### Lead contact

Requests for further information and resources should be directed to and will be fulfilled by the lead contact, Paulo R.F. Rocha (procha@uc.pt).

### Materials availability

This study did not generate new unique reagents.

### Data and code availability

• All the data reported in this paper will be shared by the lead contact upon request.
• This paper does not report original code.
• Any additional information required to reanalyze the data reported in this paper is available from the lead contact upon request.

## Acknowledgments

The 10.13039/501100019370Foundation for Science and Technology (Portugal) supported André Caeiro’s fellowship (SFRH/BD/137819/2018). This work was carried out at the R&D Unit Center for Functional Ecology—Science for People and the Planet (CFE), with reference UIDB/04004/2020, financed by FCT/MCTES through national funds (PIDDAC). P.R.F.R. acknowledges the support and funding from the 10.13039/501100000781European Research Council (ERC) under the European Union’s Horizon 2020 research and innovation programme (grant agreement no.947897).

## Author contributions

J.C. and P.R.F.R. contributed to the conception and design of the study. A.C. performed the experimental work. A.C. and P.R.F.R. analyzed the data; A.C. and P.R.F.R. wrote the manuscript. J.C. and P.R.F.R. revised the manuscript. All authors reviewed and approved the final version of the manuscript.

## Declaration of interests

P.R.F.R. is currently an editorial board member of iScience.

## STAR★Methods

### Key resources table

**Table:** 

REAGENT or RESOURCE	SOURCE	IDENTIFIER
**Biological samples**		

NEC *callus*	Lopes et al.,^33^	NA
Tamarillo *in vitro* clones	Correia et al.^9^	NA

**Chemicals, peptides, and recombinant proteins**		

Murashige and Skoog Medium including vitamins	Duchefa Biochemie	M0222

**Software and algorithms**		

MATLAB	Mathworks	https://www.mathworks.com/products/matlab.html
ImageJ	ImageJ	https://imagej.org

### Experimental model and study participant details

#### *In vitro* culture

Leaf segments from previously *in vitro* established clones^33^ were used to induce callus. For SE induction, the leaf segments were aseptically removed from the plantlets and cultured on Murashige and Skoog medium^34^ supplemented with 26 mM of sucrose and 20 μM of picloram and jellified with 2.5 g/L of PhytagelTM. After 14 weeks, the NEC samples were retrieved and multiplied from the initial masses.

The EIS assays were made on the standard induction medium, with picloram supplementation (auxin-rich medium) as well as without picloram supplementation (auxin-free medium). The pH of all the culture media used in this work was adjusted to 5.7 before adding the gelling agent. The culture media was then autoclaved at 121°C for 20 min.

Additionally, the growth profile of NEC was measured. Approximately 20 mg of NEC callus was transferred to either auxin-rich or auxin-free medium. Callus mass was measured at 24 h intervals, for 7 days, by aseptically removing the callus to pre-weighed Petri dishes and returning the masses to the same culture medium. The growth assays were made in quadruplicate in 24 Corning® Multiple Well Plates with 1 ml of culture medium (approximately the same volume used in the EIS assay). At 0, 72 and 168 hours, a sample was removed for viability staining with fluorescein diacetate.

### Method details

#### Microscopic analysis

NEC samples grown in either-auxin rich or auxin-free medium were observed by bright-field microscopy and Scanning Electron Microscopy (SEM) at time 0, 72 and 168 hours. For optical observation, the samples were mounted on glass slides and coverslips, coloured with acetocarmine 1% and immediately examined.

For SEM observation, callus samples were removed from the medium and placed without further preparation into metallic stubs and observed in frozen conditions (253 K) at 10.0 kV, using a variable pressure scanning electron microscope (Flex SEM 1000, Hitachi, Tokyo, Japan) in backscattered electron image (BSE) mode.

For viability staining, the samples removed at the time points previously described (0, 72 and 168 hours) were mixed in a 1 mL of fluorescein diacetate solution at 2 μg/ml. The fluorescein solution was prepared immediately before use, by diluting a stock solution of fluorescein diacetate at 1 mg/ml in acetone,^35^ in a 26 mM sucrose solution (the same concentration as the culture medium). The cells and solution were vortexed, mounted on glass slides and coverslips, and immediately observed on a Axio Image Z2 microscope, with a Plan-Apochromat objective with 450-490 nm and 500-550 nm excitation and emission wavelength, respectively.

#### Electrochemical impedance spectroscopy setup

The EIS setup used here was similar to our previous works.^26^^,^^31^ The transducer comprises a glass substrate where a pair of circular gold electrodes were thermally evaporated through a shadow mask, on top of a 10 nm titanium adhesion layer. The gold thickness was 100 nm and the area for each circular electrode was 6 mm^2^. A mechanical cell holder was used to hold the transducer with 1 mL of culture medium and callus in aseptic conditions. The circular electrodes were electrically connected with an Au strip to a contact pad outside the cell holder. All components of the cell holder were autoclaved at 121°C for 20 min. The transducers were submerged in ethanol 70 % (v/v) for 16 hours. The electrochemical measurements were made with a Solatron SI 1260 impedance analyzer in a frequency range from 1 Hz to 1 MHz, with an AC voltage of 20 mV, at room temperature and in dark conditions. Electrochemical measurements were performed using a two-electrode system.^36^ Data was collected in 1-hour intervals for 7 days. Baselines with only medium were also collected in the same conditions. All measurements were made in triplicate.

### Quantification and statistical analysis

The impedance modulus at low frequency (10 Hz) and mass were plotted as a function of time. The rate of increase in impedance (*r*_*Z*_*)* and rate of increase in mass *(r*_*m*_) were determined with the respective coefficient of determination (R^2^).

Impedance and mass data were normalized between 0 and 1, using min-max normalization^37^ (Equation 3) to effectively compare the relative change of both independent variables.

(Equation 3) $$X_{n}=\frac{X-X_{\min}}{X_{\max}-X_{\min}}$$

Where X_n_ is the normalized value, X is the original data point and X_min_ and X_max_ are the minimum and maximum values in the data set, respectively. Furthermore, Pearson correlation test was performed in the normalized values and the correlation coefficient (Person_corr_) is presented.

All data processing steps were made on MATLAB®.

In all analysed callus, the area of the porous surfaces identified by the SEM micrographs were measured using ImageJ (https://imagej.org). During viability tests, the fluorescence of each slide was measured by the raw integrated density using the same program. Analysis of variance (ANOVA) followed by Tukey’s multiple comparison test ( p<0.05) was performed.
