# Supplementary material for: Electrochemical impedance spectroscopy as a micropropagation monitoring tool for plants: A case study of tamarillo Solanum betaceum callus
Source: iScience. 2025 Jan 14;28(2):111807. doi: 10.1016/j.isci.2025.111807 (PMC11800089; doi:10.1016/j.isci.2025.111807)
Supplement: Document S1. Figure S1 and Table S1 [file mmc1.pdf]

iScience, Volume *28*

Supplemental information

Electrochemical impedance spectroscopy as a micropropagation monitoring tool for plants: A case study of tamarillo *Solanum betaceum* callus

André Caeiro, Jorge Canhoto, and Paulo R.F. Rocha

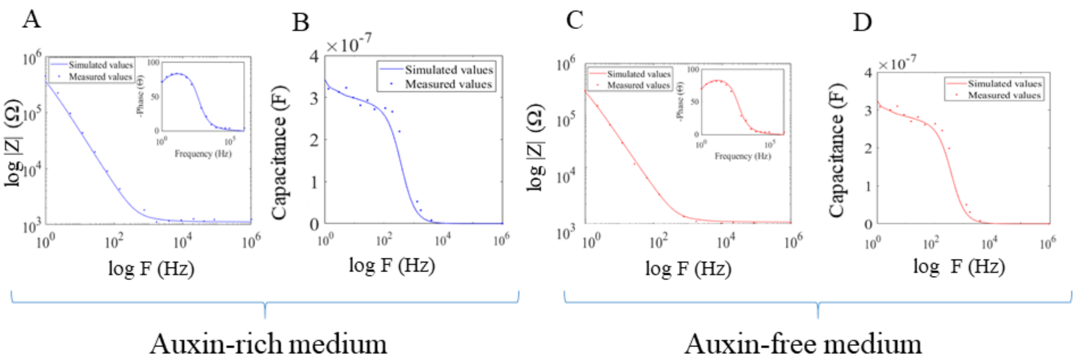

Figure S1. Impedance and Capacitance as a function of frequency, immediately after cell seeding (0 h). (A) Impedance as a function of frequency in Auxin-rich medium; inset graph: Phase angle as a function of frequency. (B) Capacitance as a function of frequency in Auxin-rich medium. (C) Impedance as a function of frequency in Auxin-free medium. Inset shows the phase angle as a function of frequency. (D) Capacitance as a function of frequency for Auxin-free medium. Measured data is represented in symbols and the simulated data is represented in solid lines and corresponds to the equivalent double RC circuit shown in Fig. 3E.

Table S1. Parameters derived from the equivalent circuit analysis.

Time (hours)	Auxin-rich medium				
	**Z (Ω)**	**Q ($\Omega^{-1}.s^{n}$)**	**$R_{CT}S$ ($\Omega.cm^2$)**	**Rsol (Ω)**	**C_{DL} ($\mu F.cm^{-2}$)**
**0**	47147.83	4.710×10^{-7}	4.46×10^{5}	592.3	13.32
**6**	47444.01	4.491×10^{-7}	4.23×10^{5}	561.9	11.45
**12**	47974.49	4.301×10^{-7}	3.04×10^{5}	547.6	11.22
**18**	53234.10	4.401×10^{-7}	3.09×10^{5}	551.6	11.17
**24**	54554.10	4.570×10^{-7}	3.47×10^{5}	568.9	11.14
**30**	58971.49	4.13×10^{-7}	2.47×10^{5}	595.9	11.07
**36**	59942.13	4.09×10^{-7}	2.34×10^{5}	601.3	11.18
**42**	60868.80	3.87×10^{-7}	2.32×10^{5}	623.9	10.45
**48**	62163.32	3.80×10^{-7}	2.28×10^{5}	626.1	10.15
**54**	63437.86	3.81×10^{-7}	2.24×10^{5}	628.2	10.10
**60**	64887.33	3.77×10^{-7}	2.24×10^{5}	625.6	9.96
**66**	65369.15	3.51×10^{-7}	2.02×10^{5}	627.1	9.38
**72**	66293.82	3.58×10^{-7}	1.97×10^{5}	632.1	9.52
**78**	67191.49	3.62×10^{-7}	1.97×10^{5}	637.2	9.62
**84**	68492.01	3.59×10^{-7}	1.99×10^{5}	642.2	9.53
**90**	69683.58	3.60×10^{-7}	2.00×10^{5}	651.2	9.53
**96**	70479.29	3.62×10^{-7}	2.00×10^{5}	663.5	9.56
**102**	71270.00	3.67×10^{-7}	2.01×10^{5}	678.6	9.67
**108**	71708.84	3.67×10^{-7}	2.01×10^{5}	692.6	9.68
**114**	72039.72	3.66×10^{-7}	2.02×10^{5}	704.1	9.68
**120**	72421.58	3.68×10^{-7}	2.03×10^{5}	711.4	9.75
**126**	72560.53	3.75×10^{-7}	2.02×10^{5}	706.8	9.93
**132**	72875.42	3.76×10^{-7}	2.03×10^{5}	683.1	9.97
**138**	72966.38	3.80×10^{-7}	2.02×10^{5}	639.9	9.97
**144**	73288.26	3.93×10^{-7}	1.95×10^{5}	633.0	9.93
**Time (hours)**	**Auxin-free medium**				
	**Z (kΩ)**	**Q ($\Omega^{-1}.s^{n}$)**	**R_{CT} S ($\Omega.cm^2$)**	**Rsol (Ω)**	**C_{DL} ($\mu F.cm^{-2}$)**

**0**	43529.81	3.91×10^{-7}	1.17×10^{4}	789.5	12.98
**6**	47147.83	4.34×10^{-7}	3.77×10^{4}	818	13.34
**12**	47444.01	4.12×10^{-7}	2.54×10^{4}	820.7	13.51
**18**	47974.49	4.03×10^{-7}	4.22×10^{4}	821.8	13.48
**24**	48528.30	3.80×10^{-7}	4.57×10^{4}	789.5	10.15
**30**	49349.88	3.81×10^{-7}	4.13×10^{4}	818	10.1
**36**	50172.48	3.77×10^{-7}	4.09×10^{4}	820.7	9.96
**42**	50773.96	3.51×10^{-7}	3.87×10^{4}	821.8	9.38
**48**	51248.66	3.58×10^{-7}	3.80×10^{4}	821.9	9.52
**54**	51935.34	3.62×10^{-7}	3.81×10^{4}	831.7	9.62
**60**	52669.69	3.59×10^{-7}	3.77×10^{4}	841.5	9.53
**66**	53302.62	3.60×10^{-7}	3.51×10^{4}	851.2	9.53
**72**	53964.96	3.62×10^{-7}	3.58×10^{4}	860.9	9.56
**78**	54656.71	3.67×10^{-7}	3.62×10^{4}	870.6	9.67
**84**	55159.81	3.67×10^{-7}	3.59×10^{4}	880.3	9.68
**90**	55574.66	3.66×10^{-7}	3.60×10^{4}	890	9.68
**96**	55852.58	3.68×10^{-7}	3.62×10^{4}	899.7	9.75
**102**	56019.94	3.75×10^{-7}	4.15×10^{4}	909.4	9.93
**108**	56471.3	3.76×10^{-7}	4.12×10^{4}	919.1	9.98
**114**	56768.49	3.80×10^{-7}	4.10×10^{4}	928.8	8.72
**120**	56991.64	3.93×10^{-7}	4.16×10^{4}	938.5	8.82
**126**	57331.43	4.15×10^{-7}	4.37×10^{4}	948.2	8.72
**132**	57474.45	4.12×10^{-7}	4.35×10^{4}	957.9	9.3
**138**	57562.69	4.10×10^{-7}	4.30×10^{4}	967.6	9.23
**144**	57665.78	4.16×10^{-7}	4.31×10^{4}	977.3	8.73
